# Innovative Nanomaterial‐Based Approaches for the Recognition of Amphetamine in Attention Deficit Hyperactivity Disorder Management

**DOI:** 10.1002/ansa.70066

**Published:** 2026-02-06

**Authors:** Ilghar Zeinaly, Ghazal Koohkansaadi, Majid Hassanzadeh‐Khanmiri, Ahmad Mobed, Saeid Charsouei, Arash Mohagheghi

**Affiliations:** ^1^ Department of Immunology School of Medicine Tabriz University of Medical Sciences Tabriz Iran; ^2^ Department of Psychology University of New Hampshire Durham New Hampshire USA; ^3^ Department of Physiology School of Medicine Tabriz University of Medical Sciences Tabriz Iran; ^4^ Tabriz University of Medical Sciences Tabriz Iran; ^5^ Department of Neurology Faculty of Medicine Tabriz University of Medical Sciences Tabriz Iran; ^6^ Research Center of Psychiatry and Behavioral Sciences Tabriz University of Medical Sciences Tabriz Iran

**Keywords:** amphetamine, attention deficit hyperactivity disorder, electrochemical sensors, nanomaterials, substance abuse monitoring

## Abstract

The rising incidence of amphetamine misuse, particularly in the context of attention deficit hyperactivity disorder treatment, underscores the urgent need for sensitive and effective detection methods. This review examines innovative nanomaterial‐based approaches for AMP detection, emphasizing their advantages over conventional analytical techniques. Various nanomaterials, including carbon nanotubes, graphene and metal nanoparticles, have been utilized to enhance the sensitivity and selectivity of detection methods such as electrochemical sensors, surface‐enhanced Raman spectroscopy and fluorescence‐based assays. The distinctive properties of nanomaterials, including high surface area, conductivity and biocompatibility, enable the development of rapid and reliable detection systems. This paper discusses recent advancements in nanomaterial synthesis, functionalization and integration into detection platforms, along with the challenges and future directions in this field. By harnessing the potential of nanotechnology, these innovative approaches aim to improve the accuracy and efficiency of amphetamine detection, thereby enhancing the monitoring and management of substance abuse, particularly in individuals with attention deficit hyperactivity disorder.

## 1 Introduction

The rise in amphetamine (AMP) abuse has emerged as a significant public health concern, prompting urgent efforts to develop effective detection methods [1, 2]. AMP, known for their stimulant properties, are often misused for recreational purposes, leading to serious health risks and societal implications [1, 2]. Traditional analytical techniques are effective but often lack sensitivity, selectivity and rapidity. This limitation highlights the need to explore new approaches that can improve detection capabilities [3, 4]. Recent advancements in nanotechnology have opened new avenues for the development of innovative detection systems [3, 4]. Nanomaterials, such as carbon nanotubes, graphene and metal nanoparticles, possess unique properties that make them ideal candidates for improving the performance of detection methods [5, 6]. Their high surface area, outstanding conductivity and biocompatibility allow for the development of sensitive and selective sensors capable of detecting AMP at lower concentrations than conventional methods [5, 6]. This review aims to provide a comprehensive overview of the current state of nanomaterial‐based detection methods for AMP. It will discuss various detection platforms, including electrochemical sensors, surface‐enhanced Raman spectroscopy (SERS) and fluorescence‐based assays, highlighting the advantages of integrating nanomaterials into these systems. In addition, the paper will discuss recent advancements in nanomaterial synthesis and functionalization, the challenges within the field and potential future directions. By leveraging the benefits of nanotechnology, these innovative approaches promise to significantly improve the accuracy and efficiency of AMP detection, ultimately supporting effective monitoring and management of substance abuse.

## 2 What is AMP?

**FIGURE 1:**
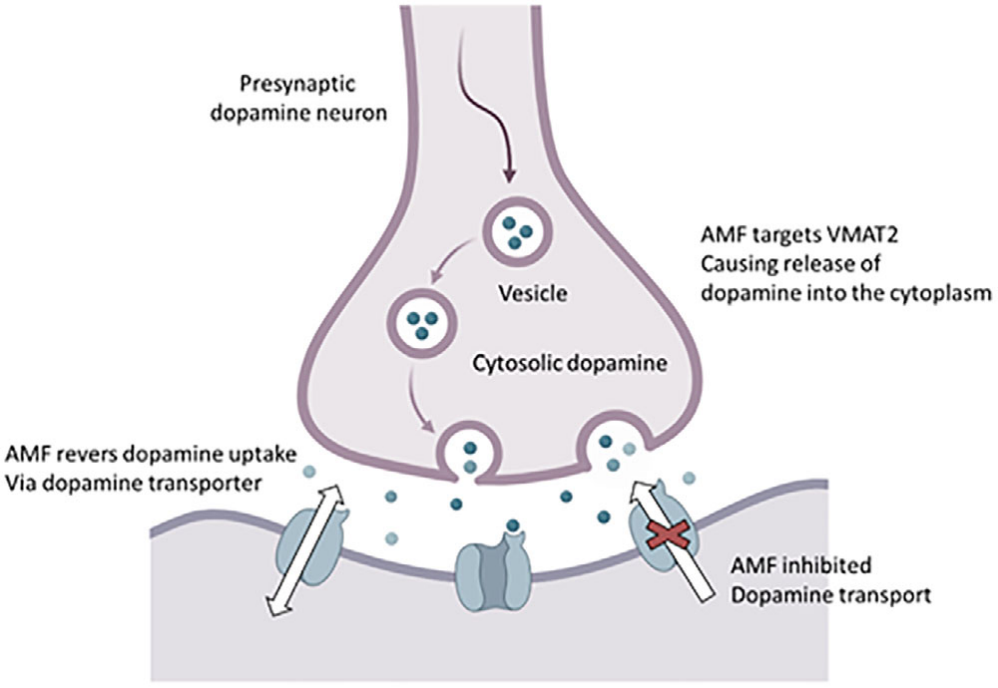
Mechanisms of action of an AMP at the dopamine synapse. This illustration depicts a presynaptic dopamine neuron where AMF targets the vesicular monoamine transporter, leading to the release of dopamine into the synaptic cleft. The dopamine transporter is inhibited by AMF, affecting dopamine reuptake and increasing cytosolic dopamine levels. This process is crucial for understanding how AMF influences dopamine signalling in the context of disorders such as ADHD. Adapted from ref. [14].

Psychostimulants, including AMP, cocaine and cathinones, are sympathomimetic substances that affect both the central nervous system (CNS) and peripheral nervous system (PNS) in ways akin to adrenaline and noradrenaline [7, 8]. Although some of these compounds are prescribed for conditions like attention‐deficit/hyperactivity disorder (ADHD), obesity and narcolepsy, their misuse can result in serious health and social consequences [7, 8]. The latest World Drug Report reveals that AMP‐type stimulants (ATS), including AMP, methamphetamine (MAMP) (often called ‘crystal meth’), 3,4‐methylenedioxymethamphetamine (MDMA, known as ‘ecstasy’ or ‘Molly’) and cathinones, are the third most commonly misused drugs worldwide, following cannabis and opioids [9, 10]. In 2020, approximately 34 million individuals reported using AMP and METH, while around 20 million consumed MDMA. The synthesis of AMP was first achieved by chemist Lazăr Edeleanu in the 1880s, although he did not conduct pharmacological studies at that time [9, 10]. Clinically, the effects of AMP can be both acute and prolonged. Acute effects on neurotransmitter release can lead to euphoria, heightened alertness, increased libido and reduced appetite [11]. Figure 1 illustrates the role of dopamine in ADHD, highlighting the presynaptic dopamine neuron and its interaction with the dopamine transporter [11]. In ADHD, there is often an imbalance in dopamine levels, which can affect attention and impulse control. The diagram shows how a substance, referred to as AMF, targets the dopamine transporter, leading to the release of dopamine into the synaptic cleft [12, 13]. This process is crucial because dopamine is a neurotransmitter that plays a significant role in regulating mood, attention and behaviour. By inhibiting dopamine uptake, AMF may contribute to the dysregulation seen in individuals with ADHD, highlighting the critical role of dopamine in managing the symptoms of this condition [12, 13]. Mechanisms of action of an AMP at the dopamine synapse are illustrated in Figure 1.

As revealed, AMF works by increasing the levels of neurotransmitters, particularly dopamine and norepinephrine, in the brain. This enhancement helps improve focus, attention and impulse control in individuals with ADHD. The mechanism involves inhibiting dopamine reuptake, which increases dopamine availability in the synaptic cleft and is essential for regulating mood and behaviour. In ADHD, AMP are often prescribed as part of a comprehensive treatment plan that may include behavioural therapy. Medications like Adderall and Vyvanse, which contain AMP, have been shown to significantly reduce symptoms in many patients [15]. The effectiveness of AMP in managing ADHD symptoms is well‐documented, making them a first‐line treatment option [16, 17]. Beyond ADHD, AMP are also used to treat other conditions. For instance, they are prescribed for narcolepsy, a sleep disorder characterized by excessive daytime sleepiness and sudden sleep attacks [16, 17]. However, due to potential side effects and the risk of dependency, their use in this area is more cautious and often considered only after other options have been exhausted [18, 19]. While AMP is effective for treating ADHD and other conditions, its use must be closely monitored because of potential side effects like increased heart rate, anxiety and the risk of substance abuse [18, 19]. A thorough evaluation by a healthcare professional is essential to determine the appropriateness of AMP treatment for each individual. The importance of AMF in the treatment of various diseases underscores the need for sensitive and rapid detection methods. In conditions like ADHD and narcolepsy, timely diagnosis is crucial for effective management. Early identification allows for prompt intervention, which can significantly improve patient outcomes by enhancing focus, attention and overall quality of life. Sensitive detection methods are essential to accurately diagnose these conditions, as symptoms can often overlap with other disorders. Rapid detection ensures that patients receive appropriate treatment without unnecessary delays, minimizing the risk of complications or worsening symptoms. Moreover, quick and reliable testing can assist healthcare providers in monitoring treatment effectiveness and potential side effects, enabling them to make informed decisions about medication adjustments. Moreover, as AMP can have side effects and potential for misuse, sensitive detection is vital in ensuring that they are prescribed responsibly. This is particularly important in vulnerable populations, such as children and adolescents, where careful monitoring is necessary to balance therapeutic benefits with safety. Overall, the integration of advanced detection techniques in clinical practice is essential for optimizing the use of AMP in disease treatment. However, high doses can result in serious adverse effects, including elevated blood pressure, hyperthermia, stroke, cardiac arrhythmias, stomach cramps and tremors. Psychological issues may also arise, such as restlessness, insomnia, aggression, delusions and hallucinations. Cardiovascular complications related to AMP use can involve several heart conditions, such as cardiomyopathy. At higher doses, hypertension and tachycardia may become more pronounced due to increased adrenergic stimulation. Abrupt cessation of high‐dose AMP use can trigger physiological and psychological effects that are the opposite of its acute effects, leading to symptoms such as weakness, tension, irritability, depression, psychosis, insomnia, impaired cognitive function, behavioural despair and even suicidal tendencies.

## 3 AMP Detection Methods

AMP can be detected using various methods in biological and pharmacological samples, each with its own advantages and limitations. One of the most commonly used methods for initial screening is immunoassays. These tests are favoured for their rapid results and can detect AMP in urine, blood and saliva. However, a significant drawback is their potential to yield false positives, which necessitates confirmatory testing to ensure accuracy. For more sophisticated analysis, gas chromatography (GC) and liquid chromatography (LC) are employed [20, 21]. These techniques offer high sensitivity and specificity, making them ideal for forensic and clinical laboratories where confirmatory testing is crucial. Often, mass spectrometry (MS) is coupled with these chromatography methods to enhance detection capabilities, providing a more reliable analysis of AMP [22, 23]. Another method, thin layer chromatography (TLC), serves as a simpler approach for qualitative analysis. While TLC is cost‐effective and easy to perform, it lacks the sensitivity found in more advanced techniques, which may limit its use in certain scenarios [24, 25]. Nuclear magnetic resonance (NMR) is also a method used for analysing AMP, although it is less common in routine drug testing [26, 27]. NMR can provide detailed structural information about the compounds, which can be beneficial in specific research contexts [26, 27]. In summary, these detection methods are crucial for various applications, including clinical diagnostics, forensic analysis and drug monitoring. The choice of method typically depends on specific analysis requirements, such as the type of sample being tested, the needed sensitivity and the resources available for testing. Detection methods for AMP are summarized in Table 1.

**TABLE 1:** Detection methods for AMP: Limit of detection (LOD) and characteristics.

Method	LOD	Description	Pros	Cons	Refs.
**GC**	1–10 ng/mL	Separates compounds based on their volatility. Often coupled with mass spectrometry (MS).	High sensitivity and specificity.	Requires sample preparation and is time‐consuming.	[28]
**LC**	1–10 ng/mL	Separates compounds in liquid samples. Can be coupled with MS for enhanced detection.	Good for complex matrices.	More expensive and requires skilled personnel.	[29]
**TLC**	100–500 ng/mL	Separates compounds on a plate coated with a stationary phase.	Simple and cost‐effective.	Lower sensitivity compared to other methods.	[30]
**NMR**	10–100 µg/mL	Analyses molecular structure based on magnetic properties.	Non‐destructive and provides structural information.	Limited sensitivity and requires large sample volumes.	[31]

Despite the various methods available for detecting AMP, each comes with its own limitations and disadvantages. For instance, while immunoassays are quick and easy to perform, they can yield false positives, necessitating confirmatory testing. GC and LC offer high sensitivity and specificity but require extensive sample preparation and skilled personnel, making them more time‐consuming and costly. TLC is simple and cost‐effective but lacks the sensitivity needed for accurate detection in many cases. NMR provides detailed structural information but is limited by its sensitivity and the large sample volumes required. In response to these limitations, there has been significant progress in the development of nanoscale detection methods over the past two decades. Nanomaterial‐based methods, particularly biosensors, have emerged as a promising solution for the rapid and ultra‐sensitive detection of AMP. These biosensors leverage the unique properties of nanomaterials, such as their high surface area and reactivity, to enhance detection capabilities. They can provide real‐time results with improved sensitivity compared to traditional methods, making them suitable for various applications, including clinical diagnostics and on‐site drug testing. The integration of nanotechnology into AMP detection represents a significant advancement, addressing many of the challenges associated with conventional detection methods.

## 4 Nanomaterial‐Based Biosensors: History and Structure

**FIGURE 2:**
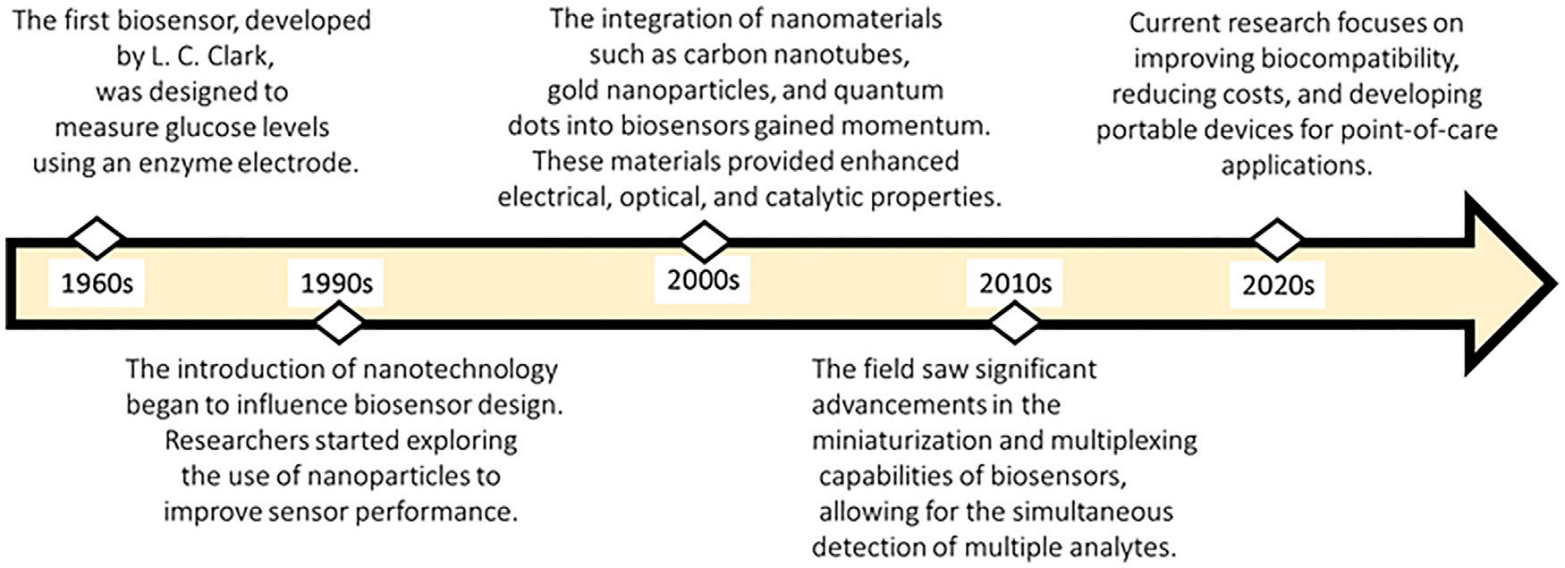
History of nanomaterial‐based biosensors. This timeline illustrates the evolution of nanomaterial‐based biosensors from the 1960s to the 2020s, highlighting pivotal advancements in technology and research. Each marked decade indicates significant breakthroughs, including the development of novel nanomaterials, integration with biological systems, and improvements in sensitivity and specificity. This visual representation underscores the transformative impact of nanotechnology on biosensing applications, particularly in fields such as healthcare and environmental monitoring. Adapted from ref. [36].

Nanomaterial‐based biosensors are analytical devices that utilize nanomaterials to detect and quantify biological substances, such as proteins, nucleic acids or pathogens [32, 33]. These biosensors combine biological recognition elements with nanomaterials to enhance sensitivity, specificity and response time. As a result, they serve as powerful tools in fields like healthcare, environmental monitoring and food safety [32, 33]. Nanomaterial‐based biosensors have emerged as a revolutionary technology in the field of diagnostics and environmental monitoring [34, 35]. These sensors leverage the unique properties of nanomaterials to enhance sensitivity, selectivity and response time. The development of nanomaterial‐based biosensors can be traced back to the early 2000s, although the concept of biosensors itself dates back to the 1960s (Figure 2) [34, 35].

**FIGURE 3:**
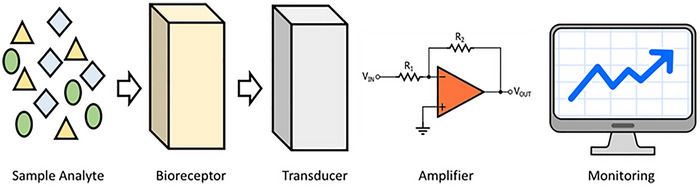
This figure depicts the workflow from raw input data, represented by various geometric shapes, through a processing unit that transforms and prepares the data. The signal amplification stage enhances the processed information, culminating in the visualization of insights on a computer monitor, showcasing trends and patterns for informed decision‐making. Adapted from ref. [41].

The structure of nanomaterial‐based biosensors consists of three main components: The recognition element, the transducer and the nanomaterials [37, 38]. The recognition element is the biological component that interacts with the target analyte. This can include enzymes that catalyse reactions, antibodies that specifically bind to antigens or nucleic acids that detect particular DNA or RNA sequences [37, 38]. The transducer converts the biological interaction into a measurable signal. It can be electrochemical, detecting changes in current or potential; optical, measuring changes in light absorption or fluorescence; or mass‐sensitive, assessing changes in mass due to binding events [39, 40]. Schematic illustration of biosensor structure presented in Figure 3.

Nanomaterials significantly enhance the performance of biosensors by providing unique properties that improve detection capabilities. For instance, carbon nanotubes boost electrical conductivity, while gold nanoparticles enhance optical signals and can be easily functionalized. Quantum dots offer specific fluorescence properties, making them ideal for targeted detection. Biosensors can be classified based on their transducer types, each with distinct characteristics, advantages and disadvantages, as outlined in Table 2. Electrochemical biosensors measure changes in electrical properties, such as current or potential, due to the interaction between the analyte and the recognition element. Common examples include amperometric and potentiometric sensors. These biosensors are known for their high sensitivity and rapid response times, along with ease of miniaturization that allows for portable applications. However, they can be susceptible to interference from other substances, leading to false readings, and they often require complex calibration processes that complicate usage. Optical biosensors detect changes in light properties—like absorption, fluorescence or reflectance when the target analyte binds to the recognition element. Examples include surface plasmon resonance (SPR) and fluorescence‐based sensors. These biosensors offer high specificity and real‐time monitoring capabilities, along with the ability to detect multiple analytes simultaneously. On the downside, they often require expensive equipment, which can limit accessibility, and their performance can be influenced by environmental conditions such as temperature and pH, potentially affecting measurement accuracy. Mass‐sensitive biosensors measure changes in mass due to analyte binding to the recognition element, often utilizing quartz crystal microbalance (QCM) technology. These sensors demonstrate high sensitivity and can provide real‐time measurements without the need for labelling. However, they are typically limited to specific applications and may not be suitable for broader use. In addition, they require careful handling to prevent damage, complicating operational procedures. This classification highlights the diversity within biosensor technology, with each type suited for different applications and environments. The assessments of pros and cons should aid in better understanding the challenges and considerations involved in deploying these technologies, ensuring responsible and informed use in various applications.

**TABLE 2:** Biosensor classification.

Transducer type	Description	Examples	Advantages	Disadvantages	Refs.
**Electrochemical**	Measures changes in electrical properties, such as current or potential, due to the interaction between the analyte and the recognition element.	Amperometric sensors, potentiometric sensors	High sensitivity: Capable of detecting low analyte concentrations. Rapid response: Quick measurements enable timely results. Miniaturization: Easy to design compact, portable devices.	Interference: Susceptible to signals from other substances, which can lead to inaccuracies. Calibration complexity: Requires intricate calibration processes and controls.	[42, 43]
**Optical**	Detects changes in light properties, such as absorption, fluorescence or reflectance, resulting from the binding of the target analyte.	Surface plasmon resonance (SPR), fluorescence‐based sensors	High specificity: Excellent for distinguishing between different analytes. Real‐time monitoring: Capable of providing immediate results. Multiplexing: Can detect multiple analytes simultaneously.	Equipment cost: Often requires expensive apparatus that may limit accessibility. Environmental sensitivity: Performance can vary with temperature and pH levels, impacting accuracy.	[44, 45]
**Mass‐sensitive**	Measures changes in mass as a result of the binding of the analyte to the recognition element, often using quartz crystal microbalance (QCM) technology.	Quartz crystal microbalance (QCM) sensors	Real‐time measurement: Provides immediate feedback without labelling. High sensitivity: Capable of detecting small mass changes.	Specificity limitations: Generally suited for particular applications, which can limit versatility. Handling care: Requires careful handling to prevent damage from environmental factors.	[46, 47]

*Note*: To improve the performance of electrochemical biosensors, recent proposals suggest modifying the working electrode (WE) using nanomaterials such as nanoparticles (NPs), carbon nanotubes (CNTs), carbon nanofibers (CNFs), quantum dots (QDs), graphene oxide (GO) and reduced graphene oxide (rGO), see Figure 4.

Electrodes play a crucial role in enhancing the performance of electrochemical sensors, with SPE, GCE and ITO electrodes being among the most widely used [49]. These electrodes can be effectively modified with a variety of nanomaterials to improve their electrochemical properties [49]. Metal nanomaterials, such as AuNPs, AgNPs and PtNPs, are particularly noteworthy due to their excellent conductivity and catalytic properties [50]. By incorporating these metal nanomaterials, the surface area of the electrodes is significantly increased, leading to higher sensitivity and faster response times in sensing applications [50]. In addition to metal nanomaterials, carbon‐based materials like graphene, graphene oxide (GO) and carbon nanotubes offer exceptional electrical conductivity and mechanical strength [51]. These materials not only enhance electron transfer rates but also provide a robust support structure for the active sensing elements. Furthermore, metal oxide nanomaterials, such as ZnO nanowires and CuO membranes, contribute to improved stability and selectivity in electrochemical reactions [51]. Quantum dots, including colloidal, graphene and QDs, can also be utilized in these electrodes to facilitate light absorption and enhance signal detection. The combination of these advanced nanomaterials within various electrode types represents a promising avenue for the development of highly sensitive and efficient electrochemical biosensors.

## 5 Biosensors in Drug Development and Analysis

In drug development, biosensors play a pivotal role by facilitating various stages, from the initial screening of potential drug candidates to the monitoring of drug interactions and pharmacokinetics [52, 53]. One of the key applications of biosensors is in high‐throughput screening, where they enable the rapid evaluation of large libraries of compounds. This capability allows researchers to identify promising drug candidates more efficiently than traditional methods, significantly accelerating the drug discovery process [54]. Biosensors also contribute to mechanistic studies, which are essential for understanding how drugs interact with their biological targets [54]. Biosensors monitor real‐time interactions between drugs and their targets, offering valuable insights into their mechanisms of action. This information helps researchers refine drug design and optimize therapeutic efficacy. Furthermore, biosensors are instrumental in pharmacokinetics, the study of how drugs are absorbed, distributed, metabolized and excreted in the body. They can track these processes in real‐time, offering critical information about a drug's behaviour within biological systems [55]. This data is vital for determining appropriate dosing regimens and understanding potential side effects. In terms of applications, cell‐based biosensors are particularly noteworthy [56]. They are used to assess drug efficacy and toxicity in cellular models, allowing researchers to evaluate how drugs affect living cells and tissues. This approach provides a more accurate representation of drug behaviour in the human body compared to traditional in vitro assays. In addition, microfluidic devices that integrate biosensors enable continuous monitoring of drug interactions in a controlled environment. These devices can simulate physiological conditions, providing a dynamic platform for studying drug effects over time [57]. Nanoscale biosensors leverage advancements in nanotechnology to enhance both sensitivity and specificity in drug detection and analysis [58]. The incorporation of nanomaterials, such as gold nanoparticles, carbon nanotubes and quantum dots, significantly improves the performance of biosensors. These materials increase the surface area available for interactions and enhance signal transduction, leading to more accurate and reliable measurements [59]. The mechanisms employed by nanoscale biosensors are diverse. SPR is one such technique that utilizes the unique optical properties of nanomaterials to detect binding events at the nanoscale [60]. This method allows for real‐time monitoring of molecular interactions, providing insights into binding kinetics and affinities [61]. Another mechanism is electrochemical detection, where nanoscale biosensors can achieve lower detection limits by amplifying electrochemical signals [62]. This capability is particularly beneficial for detecting low‐abundance biomarkers or drug molecules. The benefits of nanoscale biosensors are substantial. Their enhanced sensitivity allows for the detection of single molecules, making them ideal for early drug detection and diagnosis [62]. This level of sensitivity is crucial in identifying diseases at their onset, potentially leading to more effective treatments. In addition, miniaturizing these devices allows for their integration into portable platforms. This advancement facilitates on‐site testing and real‐time monitoring in various environments, including clinics and field studies. Biosensors represent a transformative technology in drug detection, development and nanoscale determination. Their ability to provide rapid, sensitive and specific analysis makes them invaluable in clinical diagnostics, drug development and research. As technology continues to advance, the integration of nanomaterials and innovative detection methods will further enhance the capabilities of biosensors. This progress paves the way for more effective drug monitoring and personalized medicine, ultimately improving patient outcomes and advancing healthcare.

## 6 AMP Biosensors

A sensor based on graphene‐assisted molecularly imprinted polymer nanoparticles has been developed for the detection of AMP. These nanoparticles are electroactive due to the incorporation of ferrocene into their structure. They function as specific actuators in electrochemical sensors, enabling the detection of non‐electroactive AMP through the embedded ferrocene redox probe. In a control method, the nanoparticles were covalently immobilized onto electrochemical sensors using a drop‐casting technique with silanes [63]. An electrochemical aptasensor modified with gold nanoflowers was developed for the detection of AMP. This aptasensor was created by covalently immobilizing thiolated AMP‐specific aptamers (AMP‐Apt) onto the gold nanoflowers, serving as the sensing interface. This configuration significantly increased the density of aptamers on the electrode and achieved a low limit of detection within a specific linear range, along with notable sensitivity. The influence of potentially interfering compounds on the measurement of AMP was found to be minimal for both chemical entities and biological molecules [64]. An effective EC‐SPR sensor, integrated with a molecularly imprinted strategy, was developed for the adsorption and quantitative measurement of AMPs in human urine and serum samples. The molecularly imprinted recognition system on the SPR chip was created through a straightforward one‐step electrochemical polymerization, using 3,4‐methylenedioxyphenethylamine (MDEA) as the template molecule and DA as the functional monomer [65]. A novel multiplex capacitive sensor utilizing molecularly imprinted polymers has been developed as a promising tool for detecting specific synthesis markers of AMPs in sewage water [66]. A fluorescent polymeric nanoparticle (FPN)‐based lateral flow assay (LFA) system has been developed for the rapid and sensitive detection of AMP, a significant drug of abuse today. AMP is a potent CNS stimulant known for its euphoric effects, posing a considerable threat to public health and safety. Consequently, FPNs, recognized as a promising labelling agent for on‐site and sensitive detection, are expected to make substantial contributions to the literature, as their characteristics enable accurate and reliable measurements even in complex matrices [67]. A hybrid nanozyme platform has been developed, combining GO, cationic multi‐shaped gold nanoparticles (AuNPs) and hemin, to create a novel biomimetic catalytic‐induced aptamer‐based colorimetric biosensor for the detection of AMP and MAMP. GO was electrostatically attached to cationic multi‐shaped cetyltrimethylammonium bromide (CTAB)‐coated AuNPs, resulting in a GO‐CTAB‐AuNP hybrid nanozyme that demonstrates improved catalytic activity in the presence of hemin [68]. A label‐free impedimetric sensor has been developed using cucurbit[7]uril (CB[7]) to bind ATS. This sensor incorporates three‐dimensional gold nanoparticles (3D‐AuNPs@CB[7]), characterized by advanced techniques. Their enhanced surface area and electronic conductivity enable a signal improvement of 63 times at detection limits compared to CB[7] alone, highlighting the potential of this configuration for better AMP detection, see Figure 5 [69].

**FIGURE 4:**
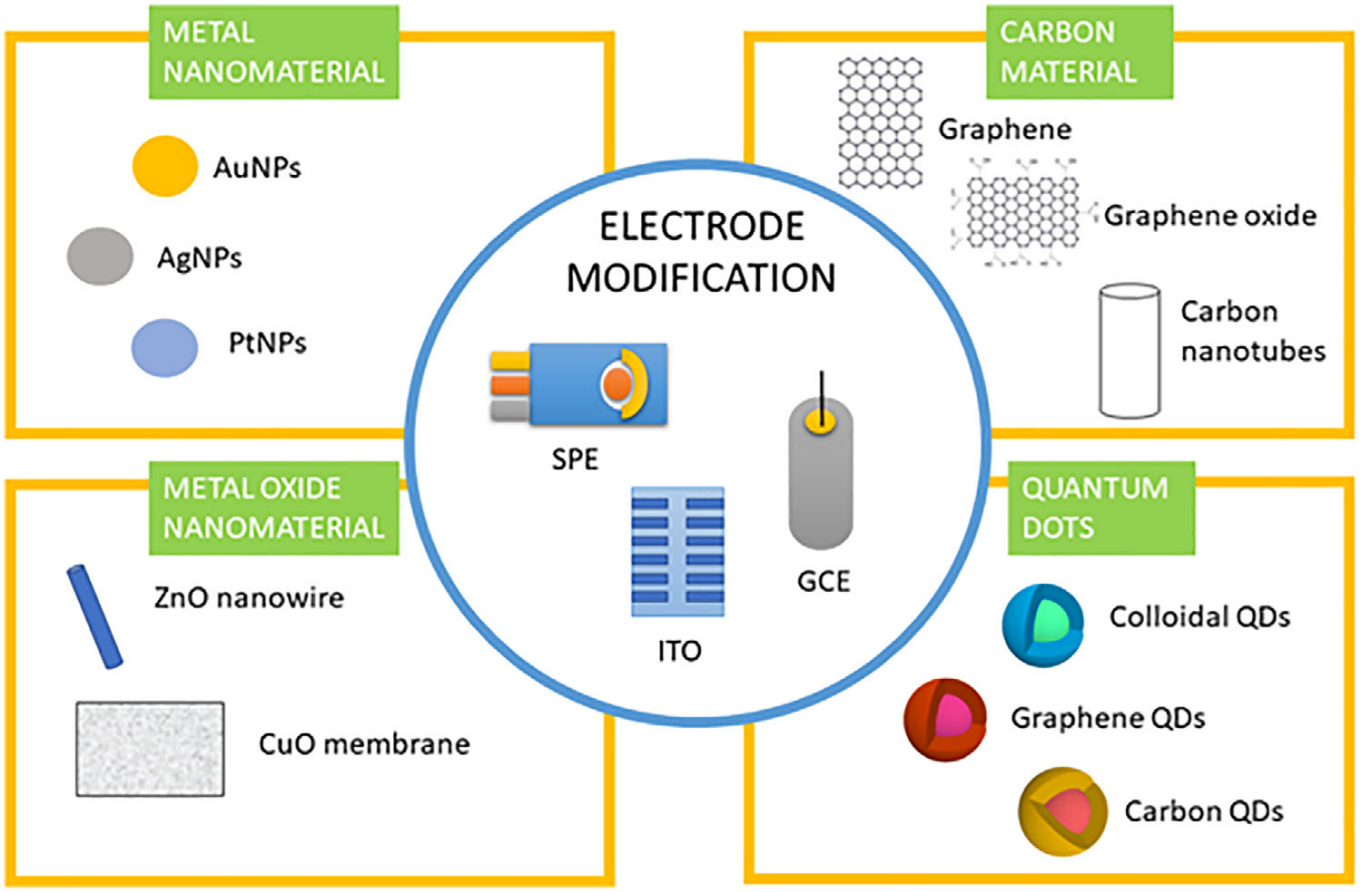
Electrodes like screen‐printed electrodes (SPE), glassy carbon electrodes (GCE) and indium tin oxide (ITO) electrodes can be enhanced with various nanomaterials. These include metal nanomaterials such as gold nanoparticles (AuNPs), silver nanoparticles (AgNPs) and platinum nanoparticles (PtNPs); carbon‐based materials like graphene, graphene oxide and carbon nanotubes; metal oxide nanomaterials such as zinc oxide (ZnO) nanowires and copper oxide (CuO) membranes; as well as quantum dots, including colloidal, graphene and carbon quantum dots (QDs) [48].

**FIGURE 5:**
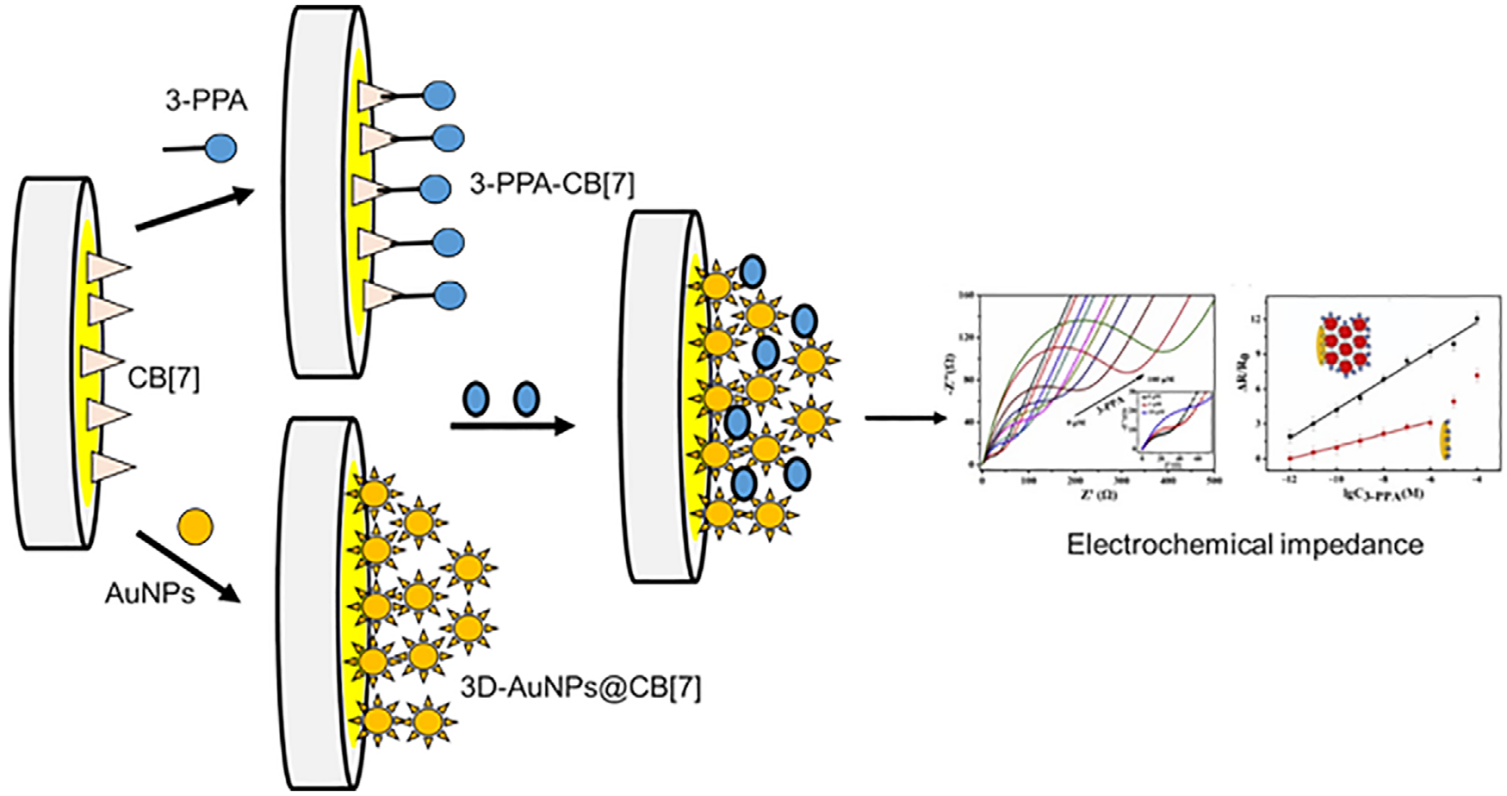
This figure illustrates the design and operation of a label‐free impedimetric sensor specifically engineered for the detection of an amphetamine‐type derivative. The sensor employs cucurbit [7]uril as a molecular receptor that enhances selectivity and sensitivity. The three‐dimensional structure of AuNPs is depicted, emphasizing their role in amplifying the sensor's electrical signal upon interaction with the target analyte. This innovative sensing platform demonstrates enhanced performance in detecting amphetamine derivatives without the need for labels, providing rapid and accurate measurements in various applications. Adapted from ref. [69].

**FIGURE 6:**
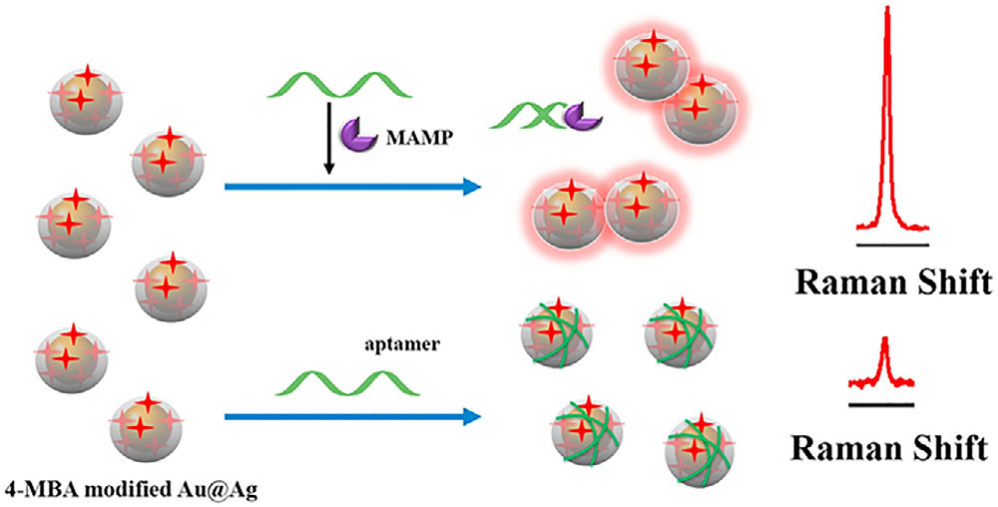
SERS‐based biosensor for identification of AMP. This figure presents the assay design for detecting MAMP using an innovative biosensor. In this setup, 4‐mercaptobenzoic acid (4‐MBA) is conjugated to gold‐silver nanoparticles (Au@Ag) through gold‐sulphur bonds, acting as a Raman reporter with a unique peak. Upon the introduction of MAMP, it binds selectively to the MAMP aptamer, displacing it from the Au@Ag surface and leading to nanoparticle aggregation. This aggregation amplifies the Raman signal of 4‐MBA due to the creation of surface‐enhanced Raman scattering (SERS) 'hot spots.' The extent of aptamer displacement and nanoparticle aggregation is directly related to the concentration of MAMP, enabling the sensor to deliver a proportional Raman signal intensity, thus resulting in an effective and straightforward MAMP biosensor [70].

A new biosensing approach has been developed for the sensitive detection of MAMP utilizing SERS. This method (Figure 6) involves manipulating the spacing between 4‐mercaptobenzoic acid (4‐MBA) labelled Au@Ag core‐shell nanoparticles (Au@Ag). To create an effective SERS substrate, Au@Ag core‐shell nanoparticles were synthesized using a seed growth method and thoroughly characterized through SEM, TEM and UV–vis spectroscopy [70].

**FIGURE 7:**
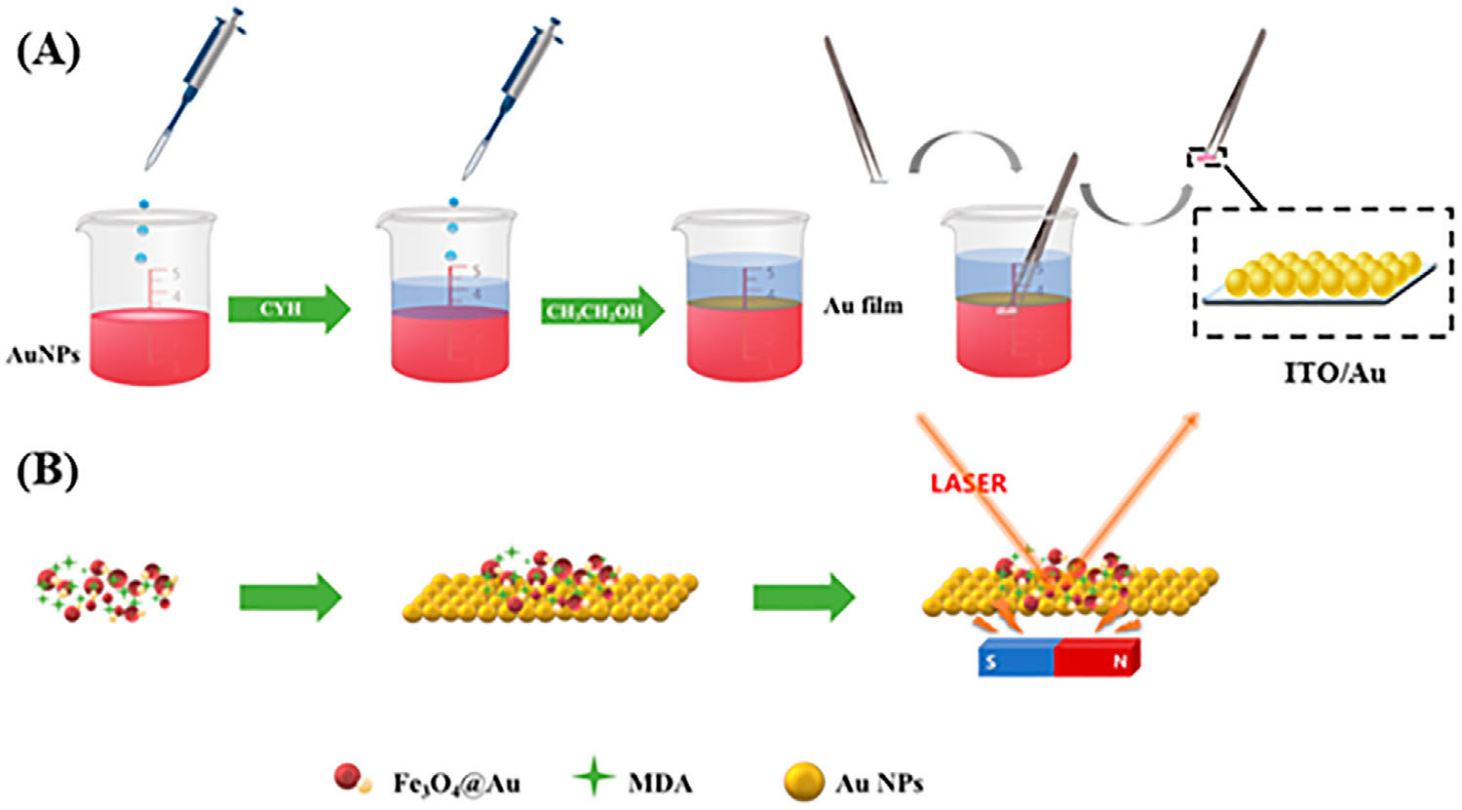
This figure illustrates the construction of a two‐dimensional substrate by transferring a self‐assembled film of gold nanoparticles (Au NPs) from a water–oil interface to indium tin oxide (ITO), referred to as ITO/Au. Concurrently, a Fe_3_O_4_@Au composite is synthesized. For on‐site detection, a sample solution containing malondialdehyde (MDA) is combined with the Fe_3_O_4_@Au. Using an external magnet, this mixture is applied to the ITO/Au substrate, forming a sandwich structure for surface‐enhanced Raman scattering (SERS) detection [71].

A two‐dimensional substrate of ITO/Au is created by transferring a gold nanoparticle film onto indium tin oxide (ITO) glass. By magnetically assembling Fe_3_O_4_@Au onto the ITO/Au substrate, a sandwich‐based SERS detection strategy is developed. The application of an external magnet allows for the retention of MDA in the hot spot regions formed between Fe_3_O_4_@Au and ITO/Au. Consequently, this method (Figure 7) demonstrates enhanced SERS sensitivity for MDA, achieving a good LOD and an acceptable linear dynamic detection range [71].

A method was established for the fabrication of silver‐gold core‐shell nanocrystals (Ag@Au SNCs). These Ag@Au SNCs serve as the fundamental material for SERS and can detect AMP at concentrations as low as 1 µg/mL. The Ag@Au SNCs demonstrate a significant SPR effect, which enhances molecular signals. The SERS spectra of ten different substances, including AMP and its analogues, revealed a pronounced peak signal [72]. A simplified approach that combines pH regulation and adsorption into a single material was developed to streamline sample preparation and improve analytical efficiency. In this study, a novel composite adsorbent made of Fe_3_O_4_/MWCNTs‐OH/CaO was created using a one‐pot grinding technique, integrating both pH adjustment and adsorption capabilities within one material. This innovation facilitated magnetic solid‐phase extraction (MSPE) without the need for pre‐adjusting the sample pH or conducting post‐desorption steps. The method was paired with liquid chromatography–tandem mass spectrometry (LC‐MS/MS) for the detection of ATS [73]. A MIP designed for the detection of AMP was synthesized. The MIP nanoparticles (nanoMIPs) were created in the presence of a template molecule. Following polymerization and the removal of the template, the MIPs were embedded with complementary cavities and functionalities. The technology described here has the potential to facilitate the rapid detection of AMP in confiscated street samples. The voltammetric sensor for AMP detection employs electroactive nanoMIPs, which are produced by incorporating ferrocene monomer into the polymeric structures, effectively serving as a transducer for the electrochemical response [74]. A rapid and dependable analytical method is vital for the on‐site detection of amphetamine‐type stimulants (ATSs). In one research, a fluorescent sensor was developed to detect the ATS derivative 3‐phenylpropylamine (3‐PPA) using a zirconium‐based metal‐organic framework (Zr‐MOF) through the fluorescence light‐up effect. The quantitative detection of 3‐PPA was accomplished in just 30 s, and the signal remained stable for up to 28 days [75]. A convenient MSPE method was developed using a newly designed and synthesized polydopamine‐functionalized core‐shell magnetic mesoporous silica (Fe_3_O_4_@nSiO_2_@mSiO_2_@PDA) nanocomposite. This approach was employed for the first time to simultaneously extract five ATSs from wastewater samples [76].

Additional information of the AMP discussed biosensors are summarized in Table 3.

**TABLE 3:** AMP biosensors.

Type	Technique	NPs	Electrode	Sample	Linear Range	LOD	Ref.
EL	MIP/DPV	NanoMIP/graphene	SPPE	Plasma	100–220 nM	68 nM	[63]
EL	DPV	Gold nanoflowers	Gold	Biological	0.1–1.0 nM	0.501 nM/µA	[64]
EL	SPR/MIP	—	NA	Urine	NA	57 nM	[65]
EL	MIP/CVs	NA	SPCE	Sewage	NA	25 µM	[66]
EL	LFAs	FNP	NA	Biological	NA	0.73 µg/mL	[67]
EL	Colorimetry	GO‐CTAB‐AuNP	NA	Biological	28.6 ng/mL	34.1 ng/mL	[68]
EL	Impedimetric	3D‐AuNPs@CB[7]	Gold	Urine and serum	10 pM to 100 µM	6.2 pM	[69]
EL	SERS/UVs	Au@Ag	NA	Real	0.5–40 ppb	0.16 ppb	[70]
EL	SERS/UVs	Fe_3_O_4_@Au	NA	Urine	5–10^5^ ng/mL	0.0685 ng/mL	[71]
EL	SERS/UVs	Ag@Au SNCs	NA	Biological	100–1000 µg/mL	NA	[72]
EL	LC‐MS/MS	Fe_3_O_4_/MWCNTs‐OH/CaO	MWCNTs	Water	0.020–0.060 ng/mL	8 ng/mL	[73]
EL	MIP	GPHOx	NA	Street	NA	NA	[74]
EL	Fluorescence	Zr‐MOF	NA	Urine	NA	NA	[75]
EL	MSPE‐UPLC‐MS/MS	Fe_3_O_4_@nSiO_2_@mSiO_2_@PDA	NA	Wastewater	1–200 ng/ L	0.5–2.5 ng/L	[76]

Abbreviation: NA, not applicable/not available.

## 7 Regulatory and Practical Considerations for AMP Biosensors

To enhance the applicability of AMP biosensors in clinical and forensic settings, it is essential to consider regulatory standards and safety assessments. Compliance with guidelines from regulatory bodies such as the FDA and EPA is critical. These biosensors must meet stringent requirements for biocompatibility and performance metrics required for clinical diagnostics. Comprehensive safety evaluations, including in vitro and in vivo studies, are necessary to assess potential biological interactions and toxicity of the nanomaterials utilized. Furthermore, developing standardized testing protocols will ensure consistency and reliability across various applications. In addition, integrating these biosensors into portable devices is crucial for practical use. Miniaturization efforts should focus on maintaining high sensitivity while ensuring that devices remain user‐friendly, facilitating home health monitoring. The incorporation of wireless technology can allow for real‐time data transmission and integration with smartphones, enhancing accessibility for end‐users. Cost‐effective and scalable production methods also warrant attention. Evaluating sustainable sourcing of nanomaterials and exploring scalable synthesis techniques, such as chemical vapour deposition and solvothermal synthesis, can facilitate mass production at reasonable costs. Collaborations with industry partners can navigate regulatory challenges and optimize production efficiency, further bolstering the practical deployment of these innovative biosensors.

## 8 Conclusion and Future Prospective

The review highlights the critical role of innovative nanomaterial‐based approaches in enhancing the detection of AMPs. The diverse range of nanomaterials, including carbon nanotubes, graphene and metal nanoparticles, has demonstrated significant advantages over traditional detection methods, particularly in terms of sensitivity and selectivity. The various techniques explored, such as electrochemical sensors, SERS and fluorescence‐based assays, showcase the potential of these nanomaterials to facilitate rapid and reliable detection systems. The data presented in Table 3 illustrates the impressive performance of different AMP biosensors, with notable LOD and linear ranges across various sample types, underscoring the advancements made in this field. Despite the promising developments, challenges remain, including the need for further optimization of nanomaterial synthesis and functionalization, along with seamless integration into user‐friendly platforms, is necessary. Addressing these challenges will be essential for translating laboratory successes into practical applications in real‐world settings. Looking ahead, there are several key areas that warrant further exploration to enhance the efficacy of AMP detection methods. Continued research into novel nanomaterials and hybrid systems could lead to even greater sensitivity and specificity in AMP detection. By exploring combinations of different nanomaterials, researchers may uncover synergistic effects that enhance performance. The development of portable detection devices utilizing these advanced nanomaterials will be crucial for on‐site testing and real‐time monitoring of AMP abuse. Such innovations could significantly improve accessibility and response times in various settings, including clinical and law enforcement environments. Establishing standardized protocols for the synthesis, characterization and testing of nanomaterial‐based sensors will be essential for ensuring reproducibility and reliability across different laboratories and applications. Encouraging collaboration between chemists, material scientists and healthcare professionals can foster innovative solutions that address the multifaceted challenges of AMP detection and substance abuse management. As these technologies advance, engaging with regulatory bodies will be important to ensure that new detection methods meet safety and efficacy standards for clinical and forensic use. By focusing on these future directions, the field can continue to leverage the unique properties of nanotechnology to improve the accuracy and efficiency of AMP detection, ultimately contributing to better monitoring and management of substance abuse.

## Author Contributions

**Ilghar Zeinaly**: investigation, data curation, writing – original draft, approval of the final manuscript. **Ghazal Koohkansaadi**: writing – original draft, writing – review and editing, resources, approval of the final manuscript. **Majid Hassanzadeh‐Khanmiri**: data curation, writing – original draft, approval of the final manuscript. **Ahmad Mobed**: conceptualization, supervision, project administration, approval of the final manuscript. **Saeid Charsouei**: corresponding author, project administration, writing – review and editing, approval of the final manuscript. **Arash Mohagheghi**: validation, formal analysis, writing – review and editing, approval of the final manuscript.

## Funding

The authors have nothing to report.

## Conflicts of Interest

The authors declare no conflicts of interest.

## Data Availability

The data that support the findings of this study are available from the corresponding author upon reasonable request.
